# GrowMatch: An Automated Method for Reconciling *In Silico*/*In Vivo* Growth Predictions

**DOI:** 10.1371/journal.pcbi.1000308

**Published:** 2009-03-13

**Authors:** Vinay Satish Kumar, Costas D. Maranas

**Affiliations:** 1 Department of Industrial and Manufacturing Engineering, The Pennsylvania State University, University Park, Pennsylvania, United States of America; 2 Department of Chemical Engineering, The Pennsylvania State University, University Park, Pennsylvania, United States of America; King's College London, United Kingdom

## Abstract

Genome-scale metabolic reconstructions are typically validated by comparing *in silico* growth predictions across different mutants utilizing different carbon sources with *in vivo* growth data. This comparison results in two types of model-prediction inconsistencies; either the model predicts *growth* when *no growth* is observed in the experiment (GNG inconsistencies) or the model predicts *no growth* when the experiment reveals *growth* (NGG inconsistencies). Here we propose an optimization-based framework, GrowMatch, to automatically reconcile GNG predictions (by suppressing functionalities in the model) and NGG predictions (by adding functionalities to the model). We use GrowMatch to resolve inconsistencies between the predictions of the latest *in silico Escherichia coli* (*i*AF1260) model and the *in vivo* data available in the Keio collection and improved the consistency of *in silico* with *in vivo* predictions from 90.6% to 96.7%. Specifically, we were able to suggest consistency-restoring hypotheses for 56/72 GNG mutants and 13/38 NGG mutants. GrowMatch resolved 18 GNG inconsistencies by suggesting suppressions in the mutant metabolic networks. Fifteen inconsistencies were resolved by suppressing isozymes in the metabolic network, and the remaining 23 GNG mutants corresponding to blocked genes were resolved by suitably modifying the biomass equation of *i*AF1260. GrowMatch suggested consistency-restoring hypotheses for five NGG mutants by adding functionalities to the model whereas the remaining eight inconsistencies were resolved by pinpointing possible alternate genes that carry out the function of the deleted gene. For many cases, GrowMatch identified fairly nonintuitive model modification hypotheses that would have been difficult to pinpoint through inspection alone. In addition, GrowMatch can be used during the construction phase of new, as opposed to existing, genome-scale metabolic models, leading to more expedient and accurate reconstructions.

## Author Summary

Over the past decade, mathematical models of cellular metabolism have been constructed for describing existing metabolic processes. The gold standard for testing the accuracy and completeness of these models is to compare their cellular growth predictions (i.e., cell life/death) across different scenarios with available experimental data. Although these comparisons have been used to suggest model modifications, the key step of identifying these modifications has often been performed manually. Here, we describe an automated procedure GrowMatch that addresses this challenge. When the model overpredicts the metabolic capabilities of the organism by predicting growth in contrast with experimental data, we use GrowMatch to restore consistency by suppressing growth enabling biotransformations in the model. Alternatively, when the model underpredicts the metabolic capabilities of the organism by predicting no growth (i.e., cell death) in contrast with available data, we use GrowMatch to restore consistency by adding growth-enabling biotransformations to the model. We demonstrate the use of GrowMatch by reconciling growth prediction inconsistencies of the latest *Escherichia coli* model with data available at the Keio database. Despite the highly curated nature of the *Escherichia coli* model, GrowMatch identified and resolved a large number of model prediction inconsistencies by taking advantage of available compilations of experimental data.

## Introduction

There are currently 700 completely sequenced genomes along with extensive compilations of data [1] assembled after decades of experimental studies on the metabolic behavior of organisms. This has enabled the reconstruction of stoichiometric models of metabolism for about twenty [2] organisms. This process began with the metabolic characterization of prokaryotic organisms such as *Escherichia coli* [1], moved to the reconstruction of eukaryotic organisms such as *Saccharomyces cerevisiae* [3] and, more recently, to the first reconstruction of the more complex *Homo Sapiens* metabolic map [4]. The completeness and accuracy of microbial metabolic reconstructions are typically assessed by comparing the model growth predictions (i.e., presence or absence) of single and/or multiple knockout mutants for a variety of substrates against experimental data [5]–[7].

**Figure 1:**
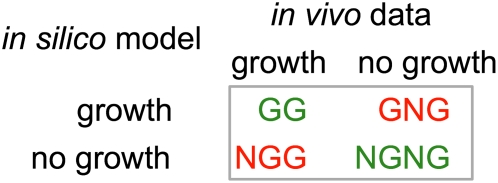
Classification of single-gene deletion mutants based on comparison of *in silico* predictions vs *in vivo* data.

As shown in Figure 1, these comparisons lead to four possible outcomes: GG when both model and experimental point at growth, GNG when the model predicts growth but the experiment does not, NGG when the model fails to predict the experimentally observed growth, and finally NGNG when both model and experiment show no growth. Cases GG and NGNG are indicative of agreement between model predictions and experimental data whereas cases GNG and NGG signify disagreement. Specifically, in GNG cases the model over-predicts the metabolic capabilities of the organism due to the use of reactions that are absent *in vivo*, down-regulation or inhibition of genes/enzymes under the experimental conditions, or absence of biomass constituents from the *in silico* biomass description. Conversely in NGG cases, the model under-predicts the metabolic capabilities of the organism due to the absence of relevant functionalities/reactions in the model. In this study, we introduce optimization-based techniques to systematically suggest modifications (conditionally add/delete reactions, restrict/expand directionalities or add/suppress uptake/secretion mechanisms for NGG/GNG inconsistencies) in genome-scale metabolic reconstructions in order to reconcile experimental and computational growth predictions across different mutants.

**Figure 2:**
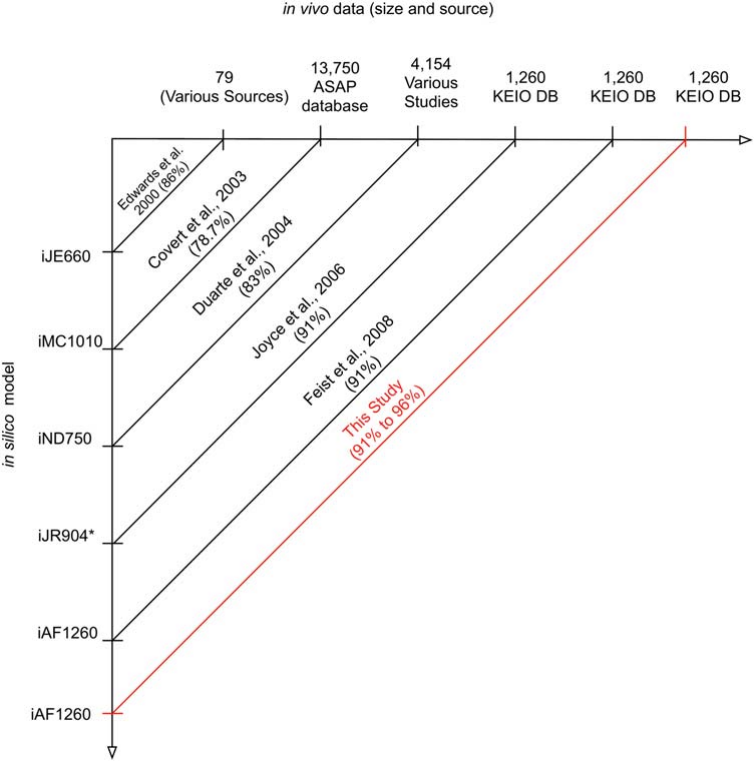
Evolution of comparisons between growth predictions of *in silico* models and observed growth in *in vivo* datasets.

The proposed method makes use of gene essentiality data sets currently available for many microorganisms [8]–[17]. For example, the Keio collection [17] catalogues the optical density (OD), under different substrate conditions, of the single gene deletion mutants of all 3,985 non essential genes in the *E. coli* K-12 BW25113. Several studies are already available that use gene essentiality data available at the Keio database and other sources to suggest targeted improvements in existing metabolic reconstructions [3], [5], [7], [18]–[20]. As seen in Figure 2, in these studies, *in silico* models of increasing complexity were successively contrasted against *in vivo* datasets of differing size to correct the predictive capabilities of the models. Recently, Joyce et al. [7] used the Keio mutant collection [17] to pinpoint conditionally essential genes *in vivo* in a glycerol supplemented minimal medium and then compared them with the corresponding *in silico* predictions to suggest improvements in the model [7]. In another study, Harrison and co-workers identified computationally predicted synthetic lethal gene deletion pairs in yeast and then proceeded to test the growth characteristics of these double deletion mutants *in vivo* [21]. While these studies have successfully used gene deletion datasets in many different contexts to pinpoint gaps in *in silico* models, the key step of resolving these gaps was performed manually.

The need to develop automated procedures to improve the accuracy of existing metabolic reconstructions has been recognized and has led to the development of a number of computational procedures. To this end, Reed et al. [22] recently described a systems based approach to modify an existing genome-scale metabolic reconstruction of *E. coli* [1] by adding new reactions that ensured growth in NGG cases by enabling *in silico* growth consistent with *in vivo* data across various carbon/nitrogen substrates. Alternatively, methods to identify and fill gaps in metabolic models based on connectivity information have also been described and applied to the genome scale models of *E. coli* and *S. cerevisiae* [23]. These studies represent only the beginning of efforts geared towards methods that automatically resolve network inconsistencies using a variety of metrics [22]–[28] ranging from unreachable metabolites, DNA microarray data and gene essentiality data. It is becoming increasingly clear that it is necessary to bring to bear all types of experimental data to achieve the aim of a high quality metabolic model.

In this paper, we supplement previous efforts [23] on identifying (i.e., GapFind) and filling (i.e., GapFill) gaps in metabolic reconstructions with an automated procedure for resolving growth prediction inconsistencies while minimally perturbing the original model. Briefly, we resolve GNG inconsistencies by converting them into NGNG one-by-one by identifying the minimal set of restrictions that need to be imposed (i.e., through reaction or transport mechanism suppression or reaction reversibility prohibition) on the model describing the GNG mutant so that biomass formation is negated (or reduced below a pre-specified cutoff). If a particular identified restriction does not invalidate any correct GG predictions then we refer to it as *global suppression* meaning that it can be imposed universally for all experimental perturbations (e.g., single gene deletion mutants and wild type). Alternatively, if an identified restriction clashes with one or more GG predictions then it is referred to as a *conditional suppression* meaning that it is imposed only in the mutant strain associated with the GNG prediction for which it is correcting.

Similarly, NGG inconsistencies are corrected one-by-one to GG by identifying the minimal set of model modifications (i.e., through reaction or transport mechanism addition or reaction reversibility allowance) that enable biomass formation (above a pre-specified cutoff). If none of these modifications affect any of the consistent NGNG cases, we refer to them as *global additions*; otherwise, we refer to them as *conditional additions*. In the next section we discuss the results obtained by applying GrowMatch to the most recent genome-scale model of *E. coli*, *i*AF1260 [20]. We note here that we can also use GrowMatch to reconcile growth prediction inconsistencies across different substrates. The *E. coli* reconstruction was chosen as the focus of this study to benchmark the ability of GrowMatch to identify model corrections even for a very well curated model. Using GrowMatch, we improved the growth prediction consistency of the *i*AF1260 model with the data available at the Keio database from 90.6% to 94.6% when considering only globally valid corrections and to 96.7% when additionally considering conditional corrections.

## Results

Here, we demonstrate the use of GrowMatch to resolve growth prediction inconsistencies between the latest *in silico* model of *E. coli* [20], and single gene-deletion mutants available at the Keio collection [17]. Specifically, we compare *in silico* growth on minimal glucose medium with the *in vivo* OD measured after 48 hours on minimal glucose. To account for the genetic differences between MG1655 (the strain used to construct the *in silico* model) and BW25113 (the strain used in the *in vivo* study), we eliminated five reactions from the *in silico* model (L-arabinose isomerase, L-ribulokinase, rhamnulokinase, L-rhamnose isomerase and rhamnulose1-phosphate aldolase) that are associated with genes (*araBAD* and *rhaBAD*) not present in the BW25113 strain. Characterizing a single gene-deletion mutant as a ‘Grow’ (G) or a ‘No-Grow’ (NG) mutant requires a cutoff for the computed (for the *in silico* model) and observed (for the *in vivo* experiment) values of growth. In this study, we adopted as the growth cutoff (i.e. 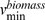 on the *in silico* side and 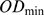 on the *in vivo* side) the one proposed in the recent study by Joyce and co-workers [7] defined as one–third of the *average* growth exhibited by all the single gene deletions under consideration. We use the same growth cutoff definition for both *in vivo* and *in silico* mutant classifications. For the *in vivo* growth classifications, we determined the growth cutoff using the data in the Keio database. For mutants with no OD measurements available, we checked the essentiality scores (available in the supplementary material for [17]) to classify them as *in vivo* essential/non-essential. Mutants with scores of greater that zero were classified as essential and those with scores less than or equal to zero were deemed non-essential. For the remaining mutants, we determined 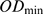 as described above and classified the gene deletion as *in vivo* essential/non-essential. Note that for computing the average OD, we assumed a value of zero OD for essential mutants with no data. As shown in Table 1, the classification of single gene-deletion mutants into one of the four categories is sensitive to the chosen cutoff (especially for the *in vivo* case).

**Table 1:** Classification of mutants depending on cutoff values chosen to distinguish between growth and no growth.

Cutoff Value	Type of Mutant			
	GNG	NGNG	NGG	GG
1%	45	112	96	1027
10%	55	135	53	1017
**33%**	**72**	**150**	**38**	**1000**
50%	107	160	28	965

Values are a percentage of *average in vivo* growth observed. In this study, we choose a 33% cutoff value based on previous studies.

**Figure 3:**
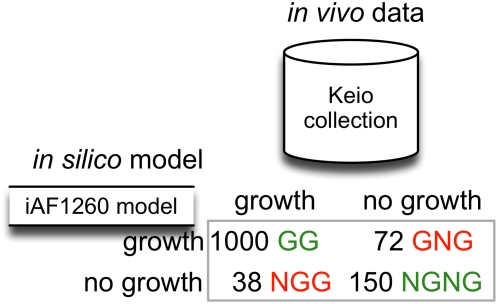
Classification of mutants based on comparison of *in silico* vs. *in vivo* data used in this study.

Figure 3 depicts the model predictions and experimental observations for growth on a minimal glucose medium. As shown, out of 1,260 single gene deletion mutants under consideration, only 110 of them have inconsistent *in silico/in vivo* growth predictions. Almost 70% of these inconsistencies are GNG implying that the *i*AF1260 model, when in error, tends to over rather than under-predict the metabolic capabilities of *E. coli*. Note that all the abbreviations used in this section are identical to the ones used in the *in silico* model of *E. coli* [20]. All the GNG and NGG mutants identified in this study are available in the supplementary material in Tables S1 and S2, respectively.

### Resolving GNG Inconsistencies

**Figure 4:**
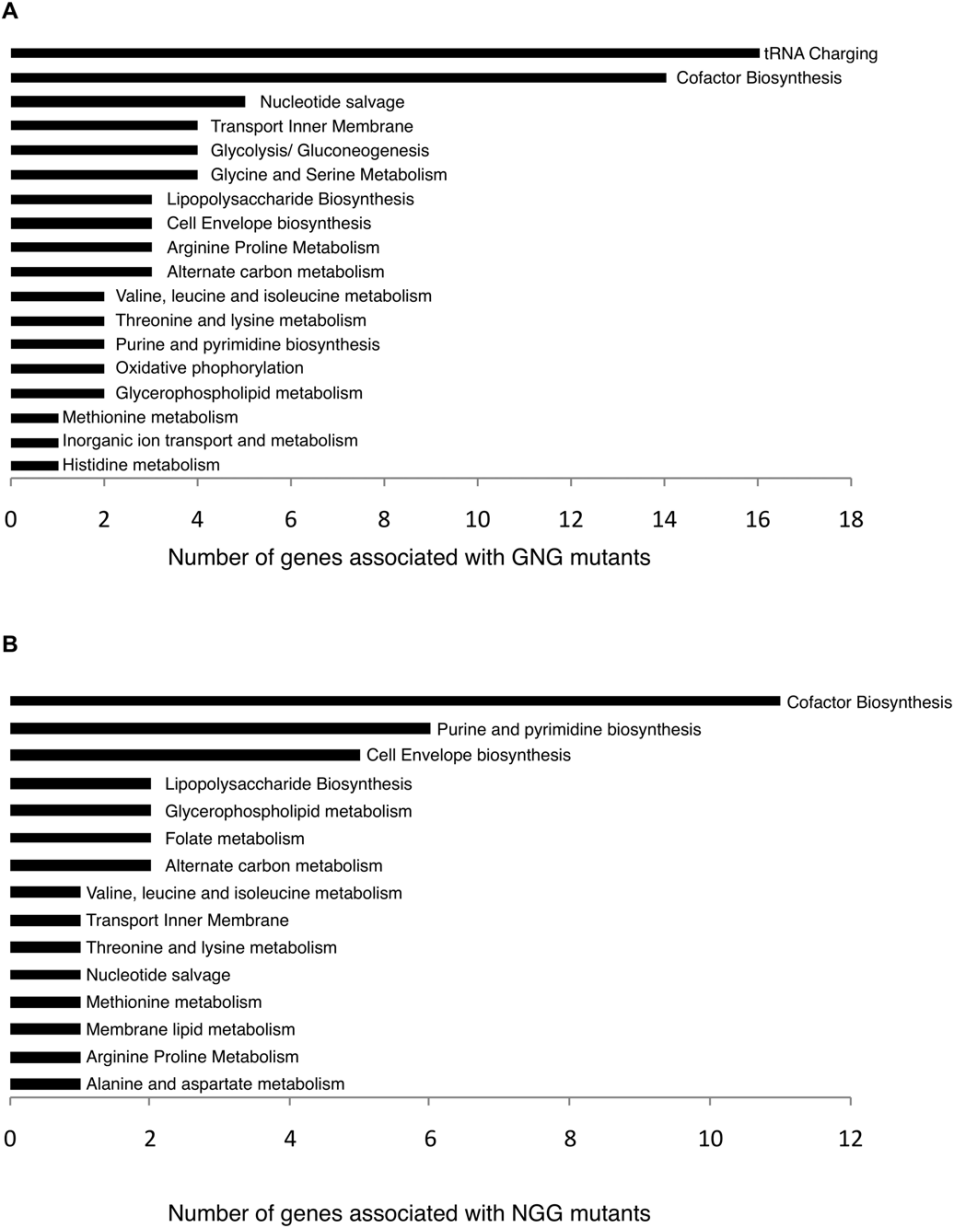
Distribution of genes associated with *inconsistent* (GNG (A) and NGG (B)) mutants across pathways in the model.

**Figure 5:**
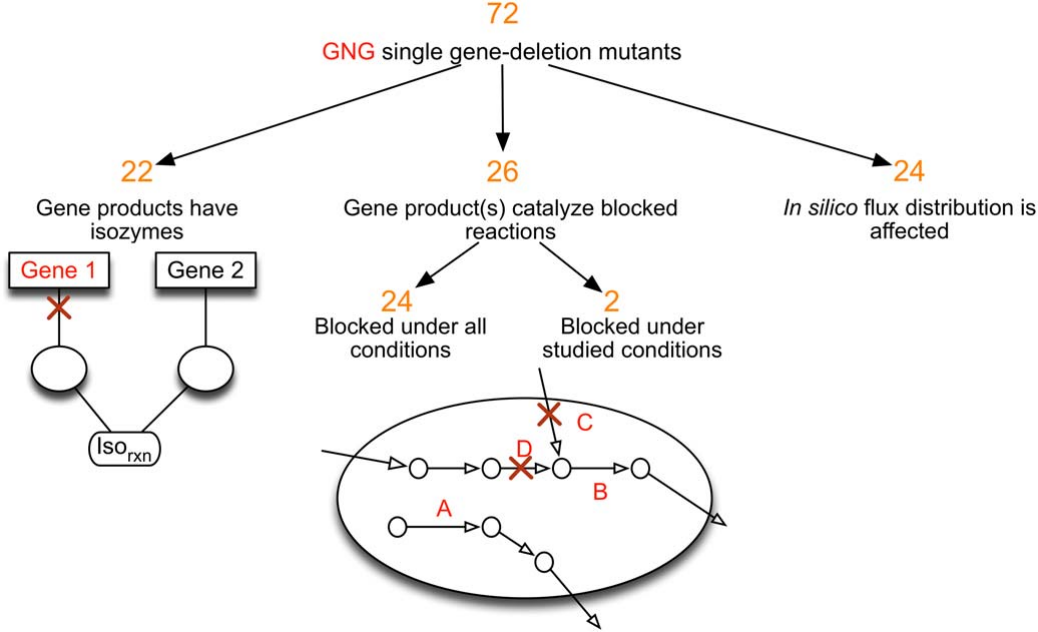
Characterization of GNG mutants identified in this study.

Figure 4A shows the distribution across pathways of the deleted genes in GNG single-gene deletion mutants. As shown, the majority of these genes are in tRNA charging and cofactor biosynthesis pathways. The presence of genes associated with GNG mutants in these pathways indicates that alternative biomass production mechanisms are implied *in silico* that are unavailable *in vivo*. Figure 5 groups these deleted genes into three categories depending on the effect of their deletion on the metabolic network. The first group (i.e., 22 GNG mutants) accounts for deleted genes whose gene-products are isozymes for reactions in the metabolic network. The presence of isozymes implies that the gene deletions do not affect the model predicted flux distributions even though *in vivo* these deletions are fatal. In these cases, we hypothesize that the *in silico* growth can be negated by simply deactivating the reaction that is catalyzed by the corresponding isozymes. In fifteen out of the twenty-two cases, the suppression of the isozymes (and the corresponding catalyzed reactions) negates growth thus converting the GNG mutants into NGNG mutants. It appears that *in vivo*, under the specific experimental conditions (aerobic glucose), the alternative isozyme does not exhibit sufficient activity to restore the activity of the deleted isozyme. Note that all these reaction suppressions are *conditional suppressions* as the reactions are essential for growth in all GG mutants. Table 2 summarizes the identified conditional suppressions. It should be noted here that these generated hypotheses may not be the only way to resolve GNG mutants associated with isozymes.

**Table 2:** Resolution of GNG mutants in which deleted genes encoding for isozymes.

GNG Mutant	Associated Essential Reaction (Pathway)
*ΔaroE*	SHK3Dr (Tyrosine, Tryptophan and Phenylalanine metabolism)
*Δcan*	HCO3E (Unassigned)
*ΔddlB*	ALAAlAr (Cell Envelope Biosynthesis)
*ΔfabZ*	12 reactions (Cell Envelope Biosynthesis)
*ΔfolA*	DHFR (Cofactor and Prosthetic Group Biosynthesis)
*ΔftsI*	MCTP1App (Murein Biosynthesis)
*ΔglnA*	GLNS (Glutamate metabolism)
*ΔilvA*	THRD_L (Valine, Leucine and Isoleucine metabolism)
*ΔmetC*	CYSTL (Methionine Metabolism)
*ΔmetE*	METS (Methionine metabolism)
*ΔmetL*	ASPK or HSDY (Threonine and Lysine metabolism)
*ΔmrdA*	MCTP1App (Murein Biosynthesis)
*ΔthrA*	ASPK or HSDY (Threonine and Lysine metabolism)
*ΔubiD*	OPHBDC (Cofactor and Prosthetic Group Biosynthesis)
*ΔyshA*	H2Otex (Transport, Outer Membrane)

We define complementary (non-complementary) isozymes as pairs of isozymes that satisfy the following two conditions: (a) at least one of the isozymes is encoded by a gene associated with a GG (GNG) mutant and (b) the isozymes catalyze an essential reaction (under aerobic glucose conditions). We checked the sequence similarity of complementary and non-complementary isozymes using the BlastP algorithm. The results are available in Table S3 Interestingly, we found that complementary isozymes have, on average, greater sequence similarity (average BLAST score ∼148 bits) than non-complementary isozymes (average BLAST score ∼69 bits).

To see if the genes that code for non-complementary isozymes are inactive under aerobic minimal glucose, we checked their expression levels. Specifically, we examined the relative expression levels for these pairs of genes (deleted gene and gene associated with non-complementing isozyme) available at Covert et al., [19]. For cases with more than one non-complementing isozyme, we checked expression data of all genes encoding non-complementing isozymes. We excluded from consideration two pairs of genes ([*thrA*, *metL*] and [*mrdA*, *ftsI*]) as all these genes are associated with GNG mutants. The 95% confidence intervals (assuming a normal distribution) for this expression data are tabulated in Table S3. In eight of the eleven cases, the deleted gene is expressed at least twice as much (using average expression as a metric) as the gene(s) associated with the non-complementing isozyme(s) (Table S3). This suggests that, in these eight cases, the genes as are expressed in very low amounts (relative to the deleted gene) in aerobic glucose conditions which indicates that the corresponding isozymes may not be at sufficient levels to insure compensation.

**Figure 6:**
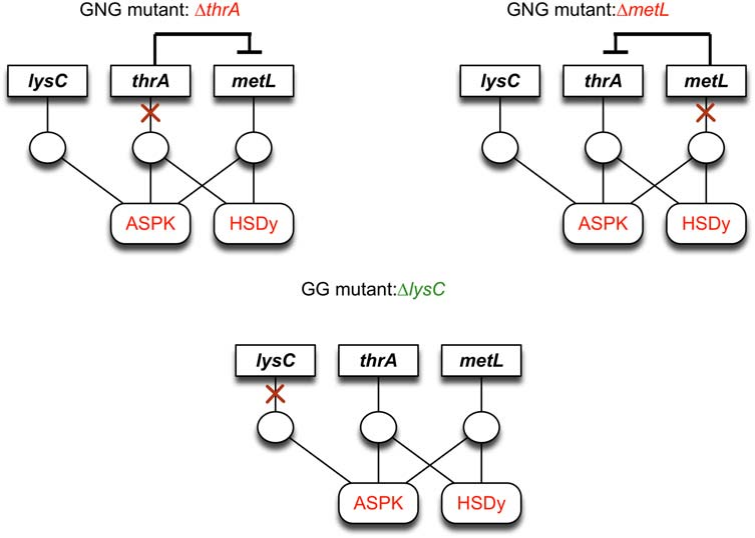
GNG mutants in which deleted genes encode for isozymes. All abbreviations are taken from the *i*AF1260 metabolic reconstruction of *E. coli*.

Figure 6 shows an example of GNG mutants associated with isozymes. Biomass formation for both single gene-deletion mutants, *ΔmetL* and *ΔthrA*, can be eliminated by suppressing any of the two associated essential reactions, aspartate kinase (ASPK) or homoserine dehydrogenase (HSDy) (see Table 2). Therefore, whenever one of the genes is deleted the other gene appears to be unable to complement the mutation and activate the two essential reactions. This implies that, as identified by GrowMatch, HSDy is inactive in both *ΔmetL* and *ΔthrA* mutants thus preventing biomass formation. Notably, HSDy is a conditional suppression as it is essential for growth in the wild-type metabolic network.

The deleted genes in the second group (i.e., 26 GNG mutants) encode for enzymes that catalyze blocked reactions in the metabolic network. Blocked reactions are defined as reactions that cannot carry any flux under given substrate conditions [29]. Twenty-four of these mutants correspond to reactions that are unconditionally blocked (i.e., for all possible substrate choices). One such example (reaction A) is shown in Figure 5. The remaining two mutants (*ΔubiG*, *ΔuxaB*) correspond to reactions that are conditionally blocked for a glucose minimal medium (e.g., reaction B in Figure 5).

GrowMatch resolved 23 of these 26 inconsistencies by suitably adding biomass components to the biomass equation. Specifically, consistency to six GNG mutants (*ΔbioB*, *ΔbioD*, *ΔbioF*, *ΔcaiT*, *ΔalsB*, *Δint*) can be restored by adding components produced by the corresponding reactions to the biomass equation (see Table S4). Modifications that restore consistency to *ΔbioB*, *ΔbioD*, *ΔbioF* are by definition *conditional modifications* since they affect the prediction for GG mutant *ΔbioA*. However, we note here that the *in vivo* OD for *ΔbioA* is very close to the cutoff (i.e., 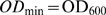 of 0.116) and it is likely that these hypotheses can be implemented as *global modifications*. The remaining mutants (*ΔcaiT*, *ΔalsB*, *Δint*) are resolved by making *global modifications*. Also, seventeen of these 26 GNG mutants correspond to reactions involved in tRNA charging reactions. GrowMatch converted these seventeen GNG mutants into NGNG mutants by modifying the biomass equation by explicitly including the charged and the uncharged tRNA molecules in place of the amino acids. For example, in the GNG mutant *ΔleuS*, the deleted reaction LEUTRS (Equation: atp+leu-L+trnaleu→amp+leutrna+ppi) is blocked. This reaction is unblocked by including leutrna (charged tRNA) and trnaleu (uncharged tRNA) as a reactant and product in the biomass equation, respectively. This restores flux through the reaction LEUTRS and converts *ΔleuS* into an NGNG mutant. We note that the consistency of these seventeen GNG mutants is restored by making *global modifications*, as adding these components to biomass does not affect any correct model predictions. For the remaining three GNG mutants, we first attempted to restore flow connectivity using (GapFill) before using GrowMatch. However, GapFill was unable to restore flow through any of these reactions by filling functionalities using reactions from the multi-organism databases of MetaCyc [30] and KEGG [31] (see Materials and Methods) thus preventing the use of GrowMatch.

The third group of GNG mutants involves deleted genes that do not encode isozymes and are not associated exclusively with blocked reactions. We used GrowMatch to identify reaction suppressions that drop the biomass production below the predefined growth cutoff. We allowed for up to *three* simultaneous suppressions per GNG mutant to ensure parsimony of correction and maintain computational tractability. As summarized in Table 3, we were able to restore consistency for eighteen of the 24 mutants. Here, ten of the identified sets of suppressions (CBMKr and OXAMTC, PPM, R15BPK, R1PK, GTHOr, GRXR. HXAND, XPPT, NACODA, R15BK) are *global* suppressions, as they did not prohibit growth in any GG mutants or wild-type strain while the remaining suppressions are *conditional*. As shown in Table 3, thirteen of the inconsistencies are resolved by suppressing one additional reaction whereas five (i.e., *ΔcarA*, *ΔcarB*, *ΔcydC*, *ΔptsI*, *ΔpyrH*) are resolved by suppressing two additional reactions in the network. Also, for ten of these GNG mutants, GrowMatch identified alternative suppression candidates (see Table 3).

**Table 3:** Resolution of GNG mutants in which flux distribution is perturbed.

GNG Mutant	Deleted Reaction(s)	Additionally Suppressed Reaction(s)
*ΔglyA*	GHMT2r	**PSP_l** or **PSERT** or **PGCD** or GLYCL
*ΔguaB*	IMPD	**XPPT** or **HXAND**
*ΔserA*	PGCD	**GHMT2r** or GLYCL
*ΔserB*	PSP_l	**GHMT2r** or GLYCL or EX_ttdcea(e)
*ΔproA*	G5SD	**NACODA**
*ΔproB*	GLU5K	**NACODA**
*ΔcarA*	CBPS	CBMKr (unassigned) and OXAMTC (unassigned)
*ΔcarB*	CBPS	CBMKr (unassigned) and OXAMTC (unassigned)
*Δadk*	13 reactions (8 with isozyme)	PPM or PRPPS or R15BPK
*ΔcydC*	CYSabc2pp, GTHRDabc2pp	(GLYAT AND GLYCL) or (AACTOOR and GLYCL)
*Δprs*	PRPPS	**PPM** or **R15BPK** or **R1PK**
*ΔgapA*	GAPD	PPS
*ΔnrdA*	RNDR1, RNDR2, RNDR3, RNDR4	TRDR or GTHOr or GRXR
*ΔnrdB*	RNDR1, RNDR2, RNDR3, RNDR4	TRDR or GTHOr or GRXR
*Δeno*	ENO	PPS
*Δpgk*	PGK	PPS
*ΔptsI*	14 reactions	FBA and TPI
*ΔpyrH*	URIDK2r	(**DURIK1 and DUTPDP**) or (**DURIPP and DUTPDP**)

Suppressions in bold are valid when the growth medium is changed from minimal glucose to minimal glycerol.

We tested the sensitivity of the identified suppressions to the growth medium by changing the medium from minimal glucose to minimal glycerol. Based on the data available in [7], all the mutants in Table 3 maintain their GNG characterization when the cell grows on minimal glycerol. As shown in Table 3, many of the identified conditional suppressions (shown in bold) needed to correct GNG predictions remain the same upon the medium change alluding to conserved regulation even under different substrates.

**Figure 7:**
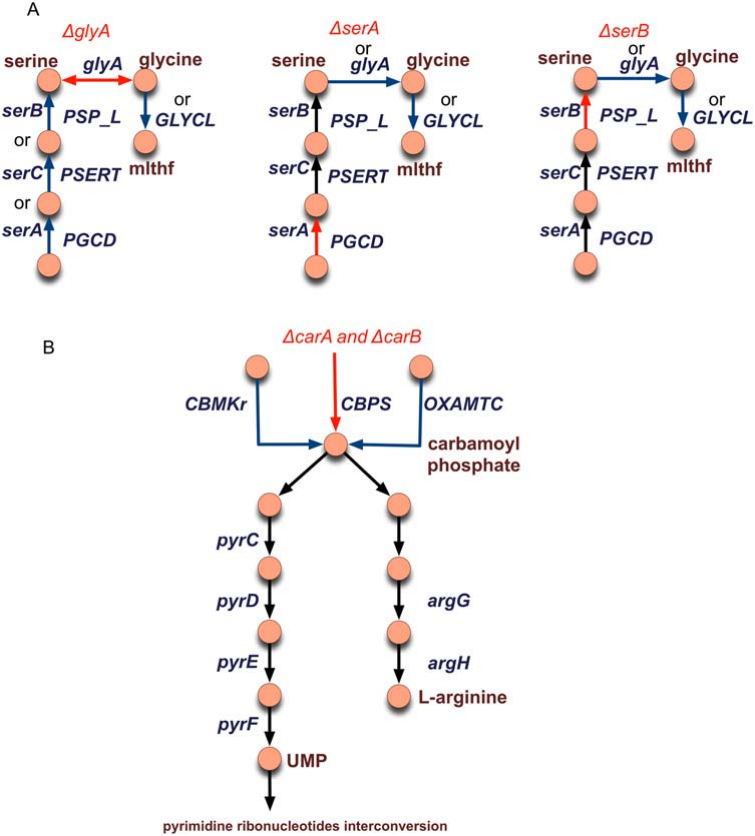
Examples showing GrowMatch's resolutions of GNG mutants where suppressions are in the same linear pathway (A) and not in the same linear pathway (B) as the deleted gene. All abbreviations are taken from the *i*AF1260 metabolic reconstruction of *E. coli*. Here reactions in blue indicate suppressions that restore consistency to the respective GNG mutant. Alternative suppressions are indicated by using the word ‘or’ above their names.

Figure 7A shows how GrowMatch restores consistency to three GNG mutants, *ΔglyA*, *ΔserA* and *ΔserB*. As shown, the gene products are involved in serine and 5,10-methylenetetrahydrofolate (mlthf) biosynthesis, both of which are essential metabolites for biomass formation. GrowMatch restores consistency in *ΔglyA* either by suppressing serine production (by deleting reactions associated with *serA*, *serB* or *serC*) or alternatively by disabling mlthf production (by suppressing the Glycine Cleavage System). In *ΔserA* and *ΔserB*, GrowMatch suggests blocking serine production by disallowing the reversibility of glycine hydroxymethyltransferase (*glyA*) (Table 3). Alternatively, as in *ΔglyA*, suppressing the Glycine Cleavage System prevents mlthf formation and thereby prohibits biomass formation. All three GNG mutants are resolved by suppressing reactions that are in the same linear pathway as the deleted reaction which is in line with evidence that genes catalyzing linear pathways of reactions tend to be co-expressed [32].

Figure 7B shows the restoration of GNG mutants, *ΔcarA* and *ΔcarB*. These genes encode for a multi-domain protein that catalyzes the reaction carbamoyl phosphate synthase (CBPS) (glutamine-hydrolysing), which is involved in the production of carbamoyl-phosphate. As shown in Figure 7B, carbamoyl phosphate (CBP) production is required for the downstream production of the biomass precursors such as L-arginine and pyrimidine ribonucleotides. GrowMatch restores consistency to these two mutants by prohibiting formation of CBP by suppressing the reactions OXAMTC and CBMKr in these mutants. In another example, GrowMatch restores consistency to the GNG mutant *ΔcydC* by suppressing GLYAT and GLYCL (Glycine Cleavage System) to prohibit biomass formation (Table 3). Note that these are conditional suppressions valid only in *ΔcydC*. Suppressing these reactions ensures that the biomass precursor metabolites, siroheme (shem) and S-Adenosyl-L-methionine (amet), are not produced in this mutant network. Closer investigation reveals that the reaction uroporphyrinogen methyltransferase, which is a reaction that consumes amet and is involved in the siroheme biosynthesis pathway, cannot carry any flux when these suppressions are carried out in *ΔcydC*. This results in no production of these biomass precursors resulting in zero biomass formation *in silico*. All the examples highlighted above lead to model modification that would have been difficult to come up with by inspection without the aid the alternatives provided by GrowMatch.

### Resolving NGG Inconsistencies

Restoring growth for the NGG predictions requires that production routes be established in the metabolic model for all 63 precursor metabolites to biomass. Figure 4B shows the location of the deleted genes across all NGG mutants. A majority of these genes are located in cofactor, cell envelope and amino acid biosynthesis pathways. As a pre-processing step, we first check if there are alternative genes that carry out the deleted function by conducting a self-BLAST search of the deleted gene against the *E. coli* K12 genome. These results are summarized in Table S5 available in the supplementary material. As seen, eight of these genes have a high sequence similarity (i.e., a protein-protein BLAST expectation value of less than 10^−13^) with other open reading frames in *E. coli*. For example, the gene *argD* whose deletion results in a NGG mutant, shares high sequence similarity with *astC* (protein-protein BLAST E-value = 5·10^−146^). Also, the gene *aspC* whose deletion results in a NGG mutant, shares a high sequence similarity (protein-protein BLAST E-value = 4·10^−94^) with *tyrB*, *which* transcribes to form a subunit of tyrosine aminotransferase. Hence, it is possible that it encodes for the activities of these genes in the respective NGG mutants *in vivo* thereby preserving growth.

We next use GrowMatch to resolve the NGG inconsistencies by adding pathways using one or more of the three mechanisms discussed previously. GrowMatch identified consistency-restoring hypotheses for 5/38 mutants. Interestingly, one NGG mutant *ΔluxS*, had alternative means of consistency restoration, one by adding reactions and the other by allowing the secretion of a metabolite. Three (including *ΔluxS*) were resolved by adding reactions from KEGG and MetaCyc [30],[31] and three (including *ΔluxS*) by allowing the secretion of metabolites from the cell into the extracellular space. None of the inconsistencies could be resolved by modifying the directionality of existing reactions in the model.

The first three NGG resolutions were corrected by adding *single* reactions from the multi-organism databases of KEGG and MetaCyc. Specifically, *ΔluxS* is corrected by adding the reaction putative adenosylhomocysteinase (from the organism *Rhizobium leguminosarum*) and *Δasd* is corrected by adding the reaction catalyzed by Protein APA1 (from the organism *Saccaromyces cerevisiae*). We note, however, that proteins catalyzing these reactions have low sequence similarity with the *E. coli* K12 genome (BLAST score = 28.1 bits with gene product of *ybcK* and 29.6 bits with gene product of *yshA* respectively) and that the validity of these hypotheses, like all those generated by GrowMatch, must be explored experimentally. Consistency in one NGG mutant (*ΔcysN*) is achieved by adding the reaction catalyzed by sulfate adenylyltransferase, the activity of which is documented in EcoCyc but was not included in the *i*AF1260 reconstruction [20],[33]. Note that adding these reactions does not disrupt any of the consistent NGNG mutants, thus these additions are referred to as *global* additions.

The other three resolutions (see Table 4) are all achieved by allowing the secretion of metabolites from the cytosol into the periplasm and out into the extracellular space. As shown, the NGG mutant *ΔfolD* is resolved by allowing the secretion of 3,4-dihydroxy-2-butanone 4-phosphate that serves as the biosynthetic precursor for the xylene ring of riboflavin. Glycolaldehyde and S-ribosyl-L-homocysteine are reactants in the reactions catalyzed by *aldA* and *luxS* respectively. To resolve the NGG mutants *ΔaldA* and *ΔluxS*, GrowMatch hypothesizes the presence of secretion mechanisms (currently absent from the model) for glycolaldehyde and S-ribosyl-L-homocysteine, respectively (Table 4). Interestingly, there is evidence that suggests that homocysteines are toxic for *E. coli* [34]. Also, as the flux value in the added secretion reaction for glycolaldehyde is very low (i.e., 2.6×10^−4^ mmol/gDW hr), it is possible that its toxic accumulation is prevented either by the (possibly non-specific) activity of a transporter that is already present or by its diffusion out of the cell.

**Table 4:** Resolution of NGG mutants by allowing secretion of metabolites.

NGG	Secreted Metabolite
**Mutant**	
*ΔaldA*	glycoaldehyde
*ΔluxS*	S-Ribosyl-L-homocysteine
*ΔfolD*	3,4-dihydroxy-2-butanone 4-phosphate

## Discussion

Here we have developed an automated procedure, GrowMatch, to resolve *in silico*/*in vivo* growth prediction inconsistencies in single gene-deletion mutants. In GNG mutants, GrowMatch restores consistency by suppressing reactions to prohibit growth. In NGG mutants, GrowMatch restores consistency by adding growth-enabling pathways. We demonstrated this procedure by reconciling the growth prediction inconsistencies between the most recent *in silico* model of *E. coli*, *i*AF1260 [20], with the *in vivo* growth data available at the Keio mutant collection [17]. Using GrowMatch, we suggested consistency-restoring hypotheses for 56/72 GNG mutants and 13/38 NGG mutants. The inconsistencies in 26 GNG mutants were resolved by carrying out conditional suppressions. In the case of NGG mutants, all the suggested modifications were global modifications. By carrying out only global modifications in wild-type *E. coli*, we were able to improve the consistency from 90.6% to 94.6%. In addition, by carrying out conditional modifications in the specific mutants, we further improve the overall consistency in growth predictions to 96.7%. Moreover, specificity has been recently proposed to be an important measure to determine the effectiveness of *in silico* simulations as a screen in computational gene essentiality predictions [35]. Notably, we improved the specificity from 67.6% to 79.3% (considering only global corrections) using GrowMatch. This value further improves to 92.8% when we also consider conditional corrections.

GrowMatch resolved eighteen GNG inconsistencies by suggesting suppressions in the mutant metabolic networks whereas fifteen inconsistencies were resolved by suppressing isozymes in the metabolic network. The remaining 23 inconsistencies corresponding to blocked genes were repaired by simply adding component(s) of the associated blocked reactions to the biomass equation (Table S4). GrowMatch suggested consistency-restoring hypotheses for five of the NGG mutants by adding functionalities to the model whereas eight inconsistencies were resolved by pinpointing alternate genes that have a high likelihood of carrying out the deleted function. Note that one NGG mutant (*ΔluxS*) had alternative means of consistency restoration.

In this study, we were able to pinpoint missing functionalities that may have been overlooked during model reconstruction. In one such example, were able to resolve a NGG mutant by adding a reaction (i.e., sulfate adenylyltransferase) with documented evidence of its being present in *E. coli* but absent in the *in silico* model *i*AF1260 [20]. Furthermore, when checking for alternative genes that restore consistency to NGG mutants, we identified possible alternative activities for *aldA* and *epd* that were not associated with them in the *i*AF1260 model (succinate semialdehyde dehydrogenase and glyceraldehyde-3-phosphate dehydrogenase, respectively). GrowMatch also resolved two NGG mutants by indirectly preventing the toxic accumulation of metabolites. Surprisingly, in the case of NGG mutants, none of the resolutions were achieved by allowing the reversibility of irreversible reactions in the model. This result is in contrast to previous results in which a large proportion of connectivity problems in the previous version of the *E. coli* genome-scale model were resolved by expanding reversibility of reactions in the model [23]. This finding may be due to the increased accuracy in the characterization of reversible reactions in the latest *E. coli* model [20] brought about by making use of ΔG values during the reconstruction process.

In line with recent explanations for GNG inconsistencies in *in silico* models [35], we find that about 33% of the GNG mutants correspond to genes associated with blocked reactions in the metabolic network. Using GapFill, we were unable to identify any flow restoring hypotheses for blocked reactions corresponding to three NGG mutants using reactions from the multi-organism databases of MetaCyc and KEGG. Also, these databases of reactions were also unable to contribute growth-enabling functionalities in 25 NGG mutants, which is likely due to the recent systematic reconciliation of the latest reconstruction of *E. coli* with data available in the MetaCyc and EcoCyc databases [30],[33]. This motivates the need to further expand the size of catalogued functionalities (e.g., the increase of experimentally determined enzyme functionalities), and also to supplement these reaction compilations with hypothetical reactions that will serve as missing links to bridge pathway gaps. There is already a large body of research focusing on deriving hypothetical reactions by iteratively changing the substrate specificity or cofactor dependence of well-characterized enzymes [36]–[40].

It is important to note that GrowMatch makes use of parsimony criteria to prioritize alternative model correcting hypotheses. Therefore, biologically relevant hypotheses that involve more than the selected maximum allowed limit of model modifications will be missed. Also, using alternate cellular objectives such as MOMA [41] or ROOM [42] instead of maximizing biomass as the objective function may help correct some GNG mutants into NGNG mutants. A recent study by Motter et al., [43] addresses this concern and defines the corresponding genes as suboptimally essential genes. It would be worthwhile to explore whether, in addition to model modifications, if more elaborate (re)definitions of objective functions [44] may be needed to improve consistency with experimental data. Furthermore, GrowMatch can also be used to reconcile growth prediction inconsistencies across various substrates. To this end, Biolog data [20] for substrate utilization (e.g., carbon, nitrogen, phosphorous and sulphur sources) can be used to propose model modifications that will ensure *in silico* growth prediction consistency with the available data.

In summary, we believe that GrowMatch, in conjunction with GapFill, are useful model-refinement tools during the reconstruction of new metabolic models or testing/curation of existing ones. In addition to the use of GrowMatch to restore growth inconsistencies for the latest *E. coli* model presented here, our group has recently used it (Suthers 2008, accepted) during the construction phase of the genome-scale metabolic model of *Mycoplasma genitalium i*PS189.

## Materials and Methods

### Definitions

First, we define the sets, parameters and variables that are common to the mathematical procedures formulated to resolve NGG and GNG inconsistencies. To this end, we define the index sets, {*i*|*i* = 1, 2… *M*},{*j*|*j* = 1, 2… *N*} and {*k*|*k* = 1, 2… *K*} that span the *M* metabolites, *N* reactions and *K* genes, respectively present in the metabolic network. Furthermore, we define the index set {*l*|*l* = 1, 2… *L*} to represent the *L in vivo* experiments under consideration. Set *KO^l^* is defined to include genes that are knocked out in experiment *l*. We define a set *Model* to include all reactions in the existing genome-scale metabolic reconstruction. We maximize the formation of biomass subject to the available substrate feed and mass balance constraints implied by the stoichiometric model. . The *in silico* predictions are then compared with *in vivo* data. *S_ij_* is the stoichiometric coefficient of metabolite *i* in reaction *j* and parameters 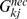, 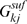 link reactions *j* to genes *k* as follows:
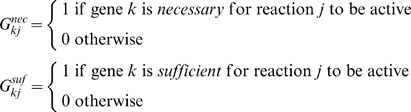



These definitions imply that if there exists two isozymes *k1* and *k2* for reaction *j* then 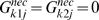 whereas 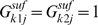. Alternatively, if the enzyme catalyzing reaction j is multimeric requiring both genes *k1* and *k2* then 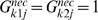 whereas 
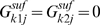
.

Upper and lower bounds, *UB_j_* and LB_j_, were chosen not to exclude any physiologically relevant metabolic flux values. The upper bound for all reactions was set to 1,000. Unless specified otherwise, the lower bound was set equal to zero for irreversible reactions and to −1,000 for reversible reactions. The flux in reaction *j* is denoted by variable *v_j_* and is restricted to vary between lower and upper bounds *LB_j_* and *UB_j_*, respectively. Using these definitions,we will now discuss the mathematical procedures developed to resolve GNG and NGG inconsistencies.

### Resolution of GNG Inconsistencies

A GNG single gene deletion mutant occurs when the model predicts growth whereas no growth is observed *in vivo*. This could be due to the erroneous presence in the model of pathways that produce biomass precursor metabolites. The aim here is to identify the minimum number of suppressions that need to be imposed for a given experiment l* corresponding to a GNG mutant to ensure that the maximum biomass formation is zero. These suppressions are carried out by either (a) restricting flux in transport/ intracellular reactions or (b) restricting the reversibility of reactions defined as reversible in the model. The description of these suppressions requires the definition of the binary variable *y_j_* to pinpoint them in the network.
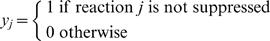



The suppressions required to ensure that the maximum biomass formation is below the imposed cut-off 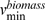 for a GNG mutant corresponding to *in vivo* experiment l* are identified by solving the following bilevel optimization problem GrowMatch:
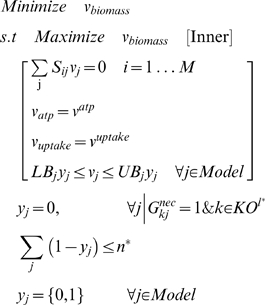



The aim of GrowMatch is to identify the minimal number of reaction suppressions needed to zero the maximum biomass formation. We do this by ensuring that there is no biomass formation even when fluxes in the network are systematically re-apportioned so as biomass formation is maximized. This leads to a *min-max* formulation. Specifically, the inner optimization problem identifies the maximum possible amount of biomass formation by redirecting metabolic fluxes subject to stoichiometry, uptake and ATP maintenance. The outer optimization problem minimizes biomass formation by choosing a pre-specified number n* of reactions in the network to suppress. A zero objective function value implies that the n* selected reaction suppressions (i.e., *y_j_* = 0) successfully prevent the network from forming biomass. This converts the GNG occurrence for *in vivo* experiment l* into NGNG restoring consistency of prediction. Alternative ways of restoring prediction consistency can be obtained by imposing successive integer cuts [45] to exclude previously identified solutions until all possible feasible solutions are exhausted. Reaction suppressions that do not inadvertently affect biomass formation in any of consistent GG prediction are referred to as *global suppressions*. On the other hand, if any of these suppressions restrict biomass production in any of the GG mutants, they are referred to as *conditional suppressions*. The identified set of suppressions (including alternative ones) is finally tested by contrasting them against literature evidence regarding the presence or absence of activity of the suppressed reaction under the experimental conditions.

For GNG mutants associated with genes encoding isozymes, we check if simply deleting the associated reaction prohibits *in silico* growth thereby restoring consistency to the mutant. For GNG mutants associated with blocked genes, we check if adding a component from the corresponding reaction to the biomass equation converts it into an NGNG mutant.

### Resolution of NGG Inconsistencies

NGG mutants are characterized by the lack of growth *in silico* despite growth *in vivo*. This means that at least one precursor metabolite in the biomass equation cannot be produced. The aim is to modify the existing genome-scale model by adding pathways so as to restore biomass production that may achieve this. To this end, we first construct a database of reactions consisting of (a) reactions from an external database of reactions, (b) irreversible reactions from the original genome-scale model with their directionalities reversed, and (c) transport reactions that enable secretion pathways for metabolites. We define the set *Database* to represent the reactions that populate this database. For the external databases of reactions, we use the multi-organism databases, MetaCyc [46] and KEGG [47], as sources of non-native functionalities. We attempt to resolve inconsistencies by adding reactions from these databases sequentially since we were unable to integrate them into a single database due to their different naming conventions. The following binary variables are defined to describe the addition of to the model.
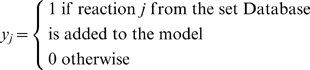



Based on these definitions, we next identify the minimal number of modifications required to correct a single NGG mutant corresponding to the *in vivo* experiment l* using the following optimization formulation GrowMatch:
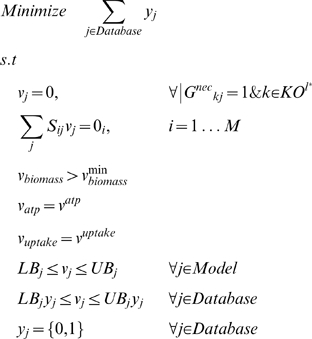



In GrowMatch, the objective function minimizes the number of modifications (addition of reactions or activation of secretion of metabolites) in the metabolic model. The first constraint enforces zero flux through reactions that are rendered absent through the elimination of the genes that are knocked out in experiment l*. The next constraint imposes stoichiometric balance on all metabolites in the model. The requirement of meeting a minimum amount of biomass, quantified by parameter 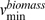, to ensure growth is imposed in the next constraint while energy requirements and uptake restrictions are imposed in the next two constraints. The final constraint ensures that if *y_j_* = 1 for a reaction *j* from the database, then there is a non-zero flux through it. The optimal solution to GrowMatch identifies the reactions that need to be added from the database and/or the metabolites that need to be secreted from the metabolic network to ensure a minimum necessary biomass production in the NGG mutant. As in the case of GNG mutants, GrowMatch can be used to identify exhaustively all sets of reactions that need to be added to resolve a particular NGG mutant using integer cuts.

We test the hypotheses generated to resolve the NGG mutant using the following two criteria. For reactions added from the database, we check the two-way protein-protein BLAST expectation value between the enzyme that catalyzes that reaction and the genome of interest (in this case *E. coli*). For irreversible reactions selected to be made reversible, we query for such evidence in the literature and also estimate the ΔG values [48] whenever available for the biotransformation in question. Finally, for secretion pathways, we query the TransportDB database [49]. A similar set of criteria were followed before in GapFill [23].

In our simulations, we set the glucose uptake rate to 10 mmol/gDW hr, ATP maintenance to 8.39 mmol/gDW and oxygen uptake rate to 15 mmol/gDW hr. We also turn off the reactions given in [20] that are down regulated in aerobic glucose conditions. We use the core biomass composition available in *i*AF1260 [20] as the *in silico* biomass description. In summary, by using the GNG and NGG GrowMatch optimization formulations, the following procedure is put forth for correcting model growth predictions:

**Step 1:** Compare *in silico* (e.g.; *i*AF1260 *E. coli* model [20]) and *in vivo* (e.g. Keio single gene-deletion collection [17]) growth predictions of all mutants. Classify mutants as GG, GNG, NGNG or NGG accordingly.
**Step 2:** Resolve GNG mutants one-at-a-time using GrowMatch by searching for suppressions (of intracellular/transport reactions and/or reversibility of reversible reactions) in restricted domains of reactions that reduce biomass production (below cutoff 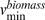). Check if these suppressions prohibit growth in any of the GG mutants. If they do not, then they are denoted as global. Otherwise, they are treated as conditional.
**Step 3:** Resolve each NGG mutant one-at-a-time by adding pathways (using external databases such as MetaCyc/KEGG [30],[31], allowing reversibility of irreversible reactions in the model, or adding secretion pathways to metabolites) to ensure biomass production using GrowMatch. Check if any of the added pathways allow for growth in any of NGNG mutants. If they do not, the additions are denoted as global. Otherwise, they are denoted as conditional.

## Supporting Information

Table S1 Blattner numbers of genes associated with GNG mutants
(0.03 MB XLS)

Table S2 Blattner numbers of genes associated with NGG mutants
(0.44 MB XLS)

Table S3 BLAST scores and expression data for complementary and non-complementary isozymes
(0.05 MB XLS)

Table S4 Sequence similarity between genes associated with NGG mutants and alternative genes in the *E. coli* genome
(0.04 MB XLS)

Table S5 Components added to *in silico* biomass equation to resolve GNG mutants associated with blocked genes
(0.02 MB XLS)
